# Can the effects of the mobilization of vulnerable elders in Ontario (MOVE ON) implementation be replicated in new settings: an interrupted time series design

**DOI:** 10.1186/s12877-019-1124-0

**Published:** 2019-04-05

**Authors:** Julia E. Moore, Barbara Liu, Sobia Khan, Charmalee Harris, Joycelyne E. Ewusie, Jemila S. Hamid, Sharon E. Straus, on behalf of the MOVE ON Collaboration, Sylvia Davidson, Terumi Izukawa, Dean Castino, Monidipa Dasgupta, Trish Fitzpatrick, Elizabeth Malloy Nantais, Alexander Nicodemo, Mary-Margaret Taabazuing, Craig Watkin, Tina Chopra, Norma McCormack, Maria Angela Tubale, Ilona Turczyn, Frederic Beauchemin, Jan-Michael Charles, Barbara Power, Vicki Thomson, Vincent G. DePaul, Diana Hatzoglou, Miranda Prince, Susan Ritchie, Bashir Versi, Natalee Elvie, Charmalee Harris, Samantha Hill, Sobia Khan, Yonda Lai, Christine Marquez, Norine Meleca, Julia E. Moore, Sharon E. Straus, Mark Wheatcroft, Maria Zorzitto, Hanan ElSherif, Jennifer Hawley, Deboura Olsen

**Affiliations:** 1 Li Ka Shing Knowledge Institute, St. Michael’s Hospital, Toronto, Ontario Canada; 2 Department of Medicine, Faculty of Medicine, University of Toronto, Toronto, Ontario Canada; 3 Regional Geriatric Program of Toronto and Sunnybrook Health Sciences Centre, Toronto, Ontario Canada; 4 Department of Health Research Methods, Evidence, and Impact, McMaster University, Hamilton, ON Canada; 5 School of Epidemiology and Public Health, University of Ottawa, Ottawa, ON Canada; 6 Children’s Hospital of Eastern Ontario, Ottawa, ON Canada

**Keywords:** Mobilization, MOVE, Mobility, Older adults

## Abstract

**Background:**

Bed rest for older hospitalized patients places them at risk for hospital-acquired morbidity. We previously evaluated an early mobilization intervention and found it to be effective at improving mobilization rates and decreasing length of stay on internal medicine units. The aim of this study was to conduct a replication study evaluating the impact of the evidence-informed mobilization intervention on surgery, psychiatry, medicine, and cardiology inpatient units.

**Methods:**

A multi-component early mobilization intervention was tailored to the local context at seven hospitals in Ontario, Canada. The primary outcome was patient mobilization measured by conducting visual audits twice a week, three times a day. Secondary outcomes were hospital length of stay and discharge destination, which were obtained from hospital decision support data. The study population was patients aged 65 years and older who were admitted to surgery, psychiatry, medicine, and cardiology inpatient units between March and August 2014. Using an interrupted time series design, the intervention was evaluated over three time periods–pre-intervention, during, and post-intervention.

**Results:**

A total of 3098 patients [mean age 78.46 years (SD 8.38)] were included in the overall analysis. There was a significant increase in mobility immediately after the intervention period compared to pre-intervention with a slope change of 1.91 (95% confidence interval [CI] 0.74–3.08, *P-value =* 0.0014). A decreasing trend in median length of stay was observed in the majority of the participating sites. Overall, a median length of stay of 26.24 days (95% CI 23.67–28.80) was observed pre-intervention compared to 23.81 days (95% CI 20.13–27.49) during the intervention and 24.69 days (95% CI 22.43–26.95) post-intervention. The overall decrease in median length of stay was associated with the increase in mobility across the sites.

**Conclusions:**

MOVE increased mobilization and these results were replicated across surgery, psychiatry, medicine, and cardiology inpatient units.

**Electronic supplementary material:**

The online version of this article (10.1186/s12877-019-1124-0) contains supplementary material, which is available to authorized users.

## Background

A rising number of older adults are admitted to acute care hospitals due to the increasing proportion of people aged 65 years and older in the general population [1]. Increased hospital admission has been accompanied by an increase in hospital-acquired disabilities in older adults related to a lack of mobilization [2–7]. Despite evidence that bed rest contributes to functional decline, older hospitalized patients spend a median of 4% of their day out of bed [8, 9]. Bed rest can result in deconditioning with loss of muscle strength, delirium, decubitus ulcers, venous thrombosis, pneumonia, increased length of stay (LOS), and admission to a nursing home [4–6, 10–13].

Early mobilization interventions may improve patients’ functional status, LOS, likelihood of returning home, and satisfaction [14–18]. Early mobilization interventions have largely been studied among patients with specific conditions, but effects are less clear when applied to any older patient in the hospital. Moreover, these studies do not provide sufficient information on how to operationalize and tailor the intervention at individual, unit, and organizational levels [19–23], making it difficult to replicate and scale up the intervention. In 2012, we developed, implemented, and evaluated an evidence-informed program to promote early mobilization in patients 65 years of age and older admitted to internal medicine units [24, 25]. We evaluated the intervention using an interrupted time series (ITS) design across 14 hospitals in Ontario, Canada. Results showed a significant increase in median weekly observed mobilization [10.56% (95% confidence interval [CI] 4.94–16.18, *P-value* < 0.001)] and a decreased LOS (3.45 days) over the study period [25]. It was unclear whether the intervention would be effective if tailored to other inpatient units. Results of a replication study would provide strong evidence that the intervention can be spread or scaled up to other inpatient units. Available literature highlights the lack of replication studies in health care, with resultant concerns about the limited research findings until replication of findings is achieved [26–28]. Similarly, there has been a lack of studies focused on scaling up interventions [29, 30]. As such, this study was developed to address these challenges. Specifically, the aim of the current study was to tailor, implement, and evaluate the effect of the early mobilization program on surgery, psychiatry, medicine, and cardiology inpatient units to determine if the study could be replicated across different settings.

## Methods

Replicating the original study design, we used a quasi-experimental ITS design to evaluate the intervention effect. Rate of mobilization, the primary outcome, was collected for 10 weeks pre-intervention, eight weeks during the intervention, and 20 weeks post-intervention over three time periods. We completed the Standards for Quality Improvement Reporting Excellence (SQUIRE) guidelines [31] and the Template for Intervention Description and Replication (TIDieR) checklist [32] [see Additional file 1]. The study methods are described in detail in a protocol paper based on the original study [24]; the methods used to replicate and scale up the study are described here. The Quality Implementation Framework was used to guide intervention implementation [33].

### Setting

The study, conducted between December 2013 and October 2014, included 16 units across seven academically-affiliated hospitals in Ontario, Canada. Units included: one cardiology unit, eight medical or medical stepdown units, one orthopedic unit, three psychiatry/behavioural units, and three surgical units.

### Readiness assessment

Original MOVE hospitals were asked to express their interest in study participation. We used a readiness assessment to select hospitals that were most ready to implement the intervention. While the readiness assessment was administered at the unit-level, the results were aggregated at the hospital-level. The readiness assessment was composed of interviews and surveys. Senior administrators, clinical managers, and/or educators in a senior leadership position on the identified units were invited to participate in a 30-min semi-structured telephone interview to discuss unit readiness including: organizational priorities; perceived benefits of patient mobilization; perceived skills and competency to lead staff in change implementation; approaches to implementation; reinforcement of improvement and monitoring; perceived barriers and facilitators to implementation; and environmental context and resources. Each interview was conducted by a trained interviewer, digitally recorded and transcribed verbatim by a transcriptionist. Two data analysts coded the data using the Framework Approach [34]. An initial review of the transcripts and notes was carried out by the analysts (i.e. *familiarization process*). A thematic framework (i.e., of central themes and categories) was developed and informed by a conceptual model of organizational readiness for change [32]. Analysts independently coded the transcripts using the thematic framework; emergent themes were incorporated to the framework throughout analysis. Analysis was conducted using NVivo 10.

Frontline staff were invited to participate in an online readiness assessment survey, the Appropriateness, Content, Facilitation Framework for Organizational Readiness Assessment [35]. Email invitations containing the survey link were sent to staff by the unit managers through existing internal listservs. The survey was designed to measure staff members’ perceptions of their unit’s/organization’s level of readiness to change by indicating their level of agreement (on a 5-point Likert scale) with statements regarding: appropriateness of the intervention; the context of the unit; and facilitators of implementation (e.g., leadership support and availability of resources). Survey data were analyzed by conducting descriptive statistics in SPSS 22.0. Using a sequential explanatory mixed methods approach [36, 37], the qualitative interview data and quantitative survey data were triangulated to identify readiness amongst leadership and frontline clinicians. To be included, hospitals needed to have strong leadership support (based on qualitative data) and some evidence of frontline buy-in (quantitative data).

### Description of the intervention

The interdisciplinary intervention, Mobilization of Vulnerable Elders (MOVE) focused on implementation of three key messages based on systematic reviews [14, 38] and feasibility: 1) patients should be assessed for mobilization status within 24 h of admission; 2) mobilization should occur at least three times a day; and 3) mobility should be progressive and scaled, individually tailored to the patient’s abilities. To support these practice changes, three core strategies were implemented: 1–2 h of education to all staff members on mobilization, up to one hour of coaching per staff member on how to implement these practice changes, and patient and family member education. Monthly audit and feedback reports were delivered to staff demonstrating preliminary audit data at multiple timepoints (twice a week and three times a day) and type of mobility as a supplementary implementation strategy to inform coaching and mobilization practices. Additional implementation strategies were tailored to local context. Specifically, at each hospital a local implementation team was created and included at a minimum, a physician lead, education coordinator and research coordinator. Centralized implementation facilitators (JEM, SK) worked with local implementation teams to tailor the intervention to unit contexts. Each participating unit identified local barriers and facilitators through focus groups with multidisciplinary frontline staff. Implementation strategies were selected and tailored by mapping barriers and facilitators to a behaviour change framework (theoretical domains framework [39]); details are presented in a previous publication [40]. The intervention mode of delivery (e.g., classroom education, in-service, e-modules, huddles, education days) was tailored to the local context and sites could use, adapt, or create new tools and resources to support the intervention. We assessed implementation activities and quality through an implementation process tool [see Additional file 2]. Primary implementation activities selected included educational meetings, staff coaching tools, education materials, and huddles. Resources developed by other hospitals from the previous evaluation were made freely available on the MOVE website (http://movescanada.ca).

### Participants

Participants included all patients aged 65 years and older admitted to one of the 16 units in the seven hospitals during the study period. Palliative patients were excluded from the study due to limited life expectancy.

### Outcomes

The primary outcome was mobilization of patients measured twice-weekly (on random days from Monday to Friday) by visual audits that took place three times per day. Patients were considered mobilized, if the visual audit identified the patient to be out of bed (sitting in chair, standing or walking with or without assistance). The primary outcome captured implementation of the key messages. The visual audits were conducted by a research coordinator at each hospital, who was trained by the central team through an online module and practice audits. Prior to data collection, the visual audit method was evaluated using three independent auditors and was found to have high inter-rater agreement (kappa = 0.83). We tested our method of visual audits against continuous rounding every 15 min for 6 h over 2 days and found it to have positive likelihood ratio 12.2 (95% CI 3.22–46.46) and negative likelihood ratio 0.06 (95% CI 0.02–0.25). The definition for mobilization in the accuracy study was that used in the audits, specifically, whether the patient was mobilized three times during the 6-h period of observation. Secondary outcomes were hospital LOS and discharge destination. We collected additional demographic and clinical data from eligible patients including age, gender, place of residence prior to admission, and admitting diagnosis. Data on the secondary outcomes were collected retrospectively from chart review and hospital decision support data. As part of the process evaluation, we documented what implementation interventions each hospital delivered in the process evaluation.

### Statistical analysis

Daily mobilization of patients, recorded from three audits per day was first summarized into proportion of patients mobilized (out of bed) at least once a day. Proportion of patients mobilized was then averaged over the two audit days, within the week the audits were recorded, to provide an estimate of average daily mobilization for a given week. This was done pre- (10 weeks, 20 assessment points), during (eight weeks, 16 assessment points), and post-intervention (20 weeks, 40 assessment points) for each hospital. To investigate the overall impact of the intervention on mobilization across all the participating sites, mobilization at each time point was first aggregated across the hospitals. The difference in the trend and level was then compared among the three periods (pre-, during and post-intervention). This was done by performing an ITS analysis using a segmented linear regression model [41]. Presence of serial autocorrelation across the different time points was assessed using the Durbin-Watson’s statistic [42]; and when statistically significant, adjustment for autocorrelation was made [43]. Hospital-level analysis was also performed to investigate site-specific assessment of the intervention and examine variation in mobilization across the sites. For the secondary outcome, daily median LOS in a given week was considered from all participating hospitals pre-, during and post-intervention. Discharge date was used to classify patients into pre-, during and post-intervention periods. The daily median LOS within a given week was averaged across all hospitals to provide an overall estimate. ITS with segmented linear regression was then performed to investigate the impact of the intervention on LOS. Site level analysis was also performed to examine site differences in outcomes. Data on discharge destination as well as other demographic and clinical variables were summarized across the different time periods. All statistical analyses were performed using the R statistical software [44] and statistical significance was determined using α = 0.05 level of significance.

## Results

### Participants and units

#### Organizational readiness

Two senior administrators, eight clinical managers, and one clinical educator were interviewed between August and September 2013, from nine hospitals that participated in the readiness assessment phase of MOVE ON+. A total of 121 surveys were completed from six of nine hospitals. Demographic information (e.g., gender, role, and number of years of clinical experience) was collected from survey participants; however, no personal identifiers were collected and therefore surveys were anonymous. Six sites had strong leadership support and frontline clinician buy-in; given that there was funding for seven hospitals, the hospitals with the next strongest leadership support was selected, even though there were no surveys from frontline clinicians [see Additional file 3].

Sixteen inpatient units participated in the intervention across seven hospitals. Each unit had between 12 and 40 beds (mean of 25.4 beds).

#### Patient mobilization

The analysis included 42,076 mobilization audits from 3098 patients [mean age 78.46 years (SD 8.38)] across six hospitals; 48.3% were female. Participant characteristics are shown in Table 1. One hospital was excluded from the overall analysis (7041 audits from 129 patients), because baseline mobility at this hospital was 91.06% compared to the overall baseline average of 58.19% across the remaining hospitals and baseline median LOS was 76.08 days compared to the overall average median LOS of 27.64; we were not aware of the high mobility rate when the site was selected. Data from this hospital were analyzed separately and site-level results are provided.

**Table 1:** Patient characteristics for the 5 sites included in the ITS analysis

		Overall	Pre	During	Post
No. of subjects (N)		3097	917	535	1645
Age [mean years (SD)]		78.46 (8.38)	78.61 (8.50)	78.75 (8.04)	78.29 (8.43)
Gender M:F [n (%)]		1562 (51.70): 1459 (48.30)	480 (52.3): 417 (45.5)	272 (50.8): 259 (48.4)	810 (49.2): 783 (47.6)
Top 5 Most Responsible Discharge Diagnoses [n (%)]	Coronary artery disease	255 (8.2)	70 (7.6)	45 (8.4)	140 (8.5)
	Congestive heart failure	171 (5.5)	50 (5.5)	31 (5.8)	90 (5.5)
	Aortic stenosis	109 (3.5)	31 (3.4)	24 (4.5)	54 (3.3)
	Acute myocardial infarction	69 (2.2)	15 (1.6)	8 (1.5)	46 (2.8)
	Urinary tract infection	67 (2.2)	21 (2.3)	12 (2.2)	34 (2.1)
Place of residence prior to admission [n (%)]	Private home, apartment or condominium	1074 (34.7)	320 (34.9)	178 (33.3)	576 (35.0)
	Acute Facility^a^	518 (16.7)	93 (10.1)	81 (15.2)	344 (20.9)
	Nursing home or Long-term care home	258 (8.3)	86 (9.4)	56 (10.5)	116 (7.0)
	Rehabilitation facility^a^	28 (0.9)	10 (1.1)	8 (1.5)	10 (0.6)
	Other (unspecified)	1214 (39.2)	407 (44.4)	211 (39.5)	596 (36.2)

^a^ when transferred from another acute or rehabilitation facility, the patient’s place of residence prior to admission is not known

### Primary outcome: patient mobilization

#### Overall results

**Fig. 1:**
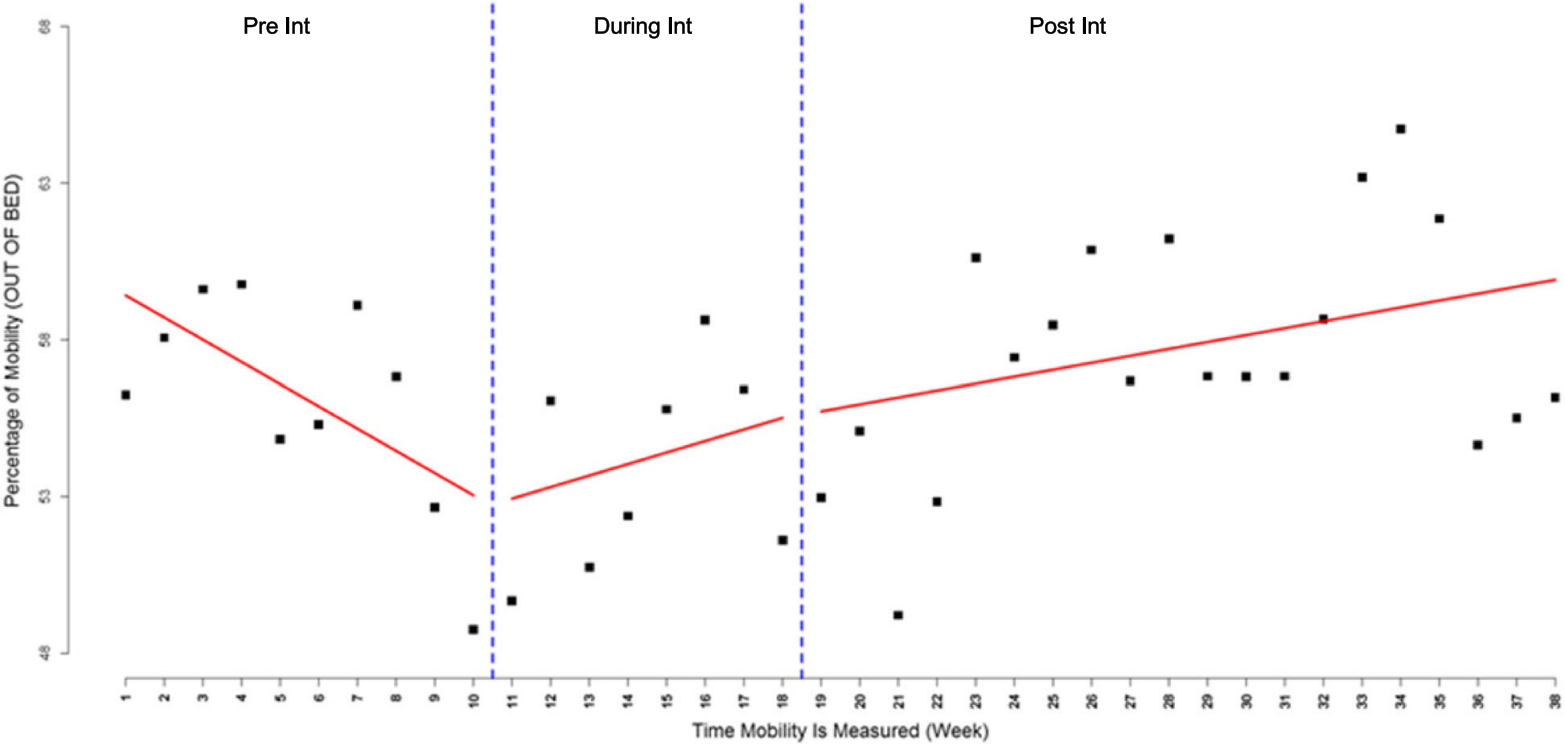
Overall weekly visual audit results for proportion of patients out of bed

Overall, combined data across the six hospitals showed an increasing trend in mobilization during the intervention phase compared to the declining trend observed in the pre-intervention period (Fig. 1), with a slope increase of 1.08 (95% CI -0.34 – 2.50, *P-value* = 0.1389). However, this difference was not statistically significant. Post-intervention there was a statistically significant increase in mobilization with a change in slope of 0.93 (95% CI 0.07–1.78, *P-value* = 0.0334) compared to pre-intervention. At the end of the study period, there was a non-significant increase in the number of patients out of bed compared to pre-intervention, where an estimated 6.9% (95% CI -1.0 – 14.8, *P-value* = 0.0924) more patients were out of bed. An estimated 2.5% (95% CI -4.7 – 9.6, *P-value* = 0.5291) more patients were out of bed per day at the intervention end compared to pre-intervention but this was not statistically significant.

**Fig. 2:**
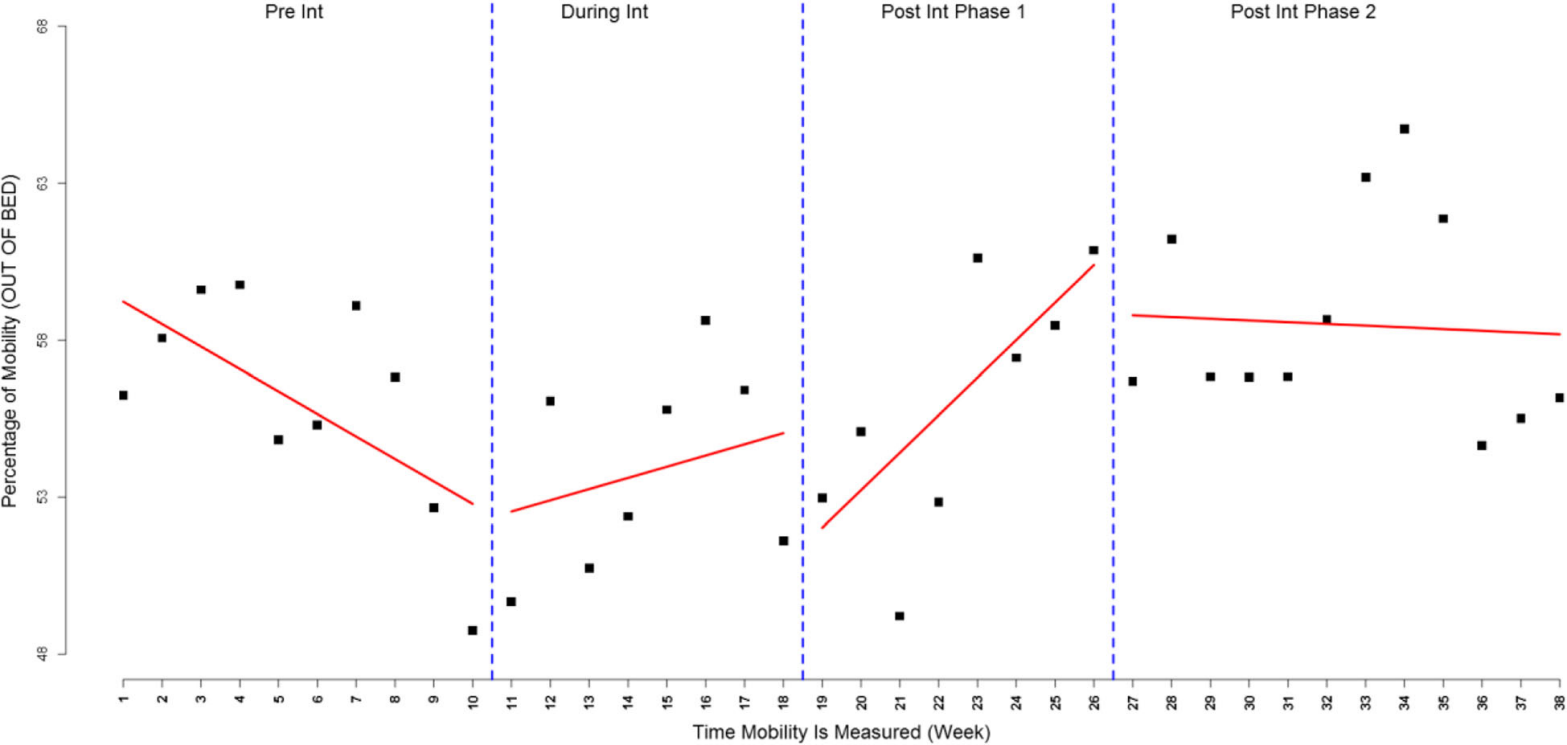
Overall weekly visual audit results for proportion of patients out of bed dividing post-intervention into two sections

While mobilization continued to increase in the first weeks of the post-intervention period, a declining trend in mobilization was observed after week 26 (Fig. 1). This pattern was observed across most of the participating hospitals, indicating a high level of immediate effect following the intervention phase followed by declines in the last weeks of the study. To investigate this further, we divided the post-intervention period into two periods and re-analyzed the data using ITS with four periods (Fig. 2). As some hospitals continued delivering implementation strategies for a couple of months after the intervention phase, we chose a cut off of 26 weeks to assess sustainability. The results showed a statistically significant increase in mobility immediately after the intervention period compared to pre-intervention with a slope change of 1.91 (95% CI 0.74–3.08, *P-value* = 0.0014). However, during the last 12 weeks, there was a statistically significant decline in the rate of mobilization compared to the early post-intervention period (slope change = − 1.25, 95% CI -2.36 – -0.13, *P-value* = 0.0281).

#### Hospital-specific results

Pre-intervention, a declining trend in mobility, which was not statistically significant, was observed across all hospitals, although their rate of decline varied. The hospital-specific results are consistent across the hospitals with respect to impact of the intervention on patient mobilization; in all six hospitals mobilization increased daily during the intervention period or immediately after the intervention compared to pre-intervention [see Additional file 4]. The majority (*n* = 4) of the hospitals observed an immediate improvement in mobilization after the intervention. In all six hospitals, an increasing trend in mobilization was observed during the first eight weeks of the post-intervention period (i.e., 16 weeks since the start of the intervention). However, mobilization started to decline after week eight of the post-intervention period in three sites. In the other three sites, mobilization increased throughout the 20 post-intervention weeks indicating sustainability of the intervention effect. For the site removed from the overall ITS analysis, there were no significant changes in mobilization across the study and the mobilization rate remained above 70% [see Additional file 5].

### Secondary outcome: length of stay

#### Overall results

**Fig. 3:**
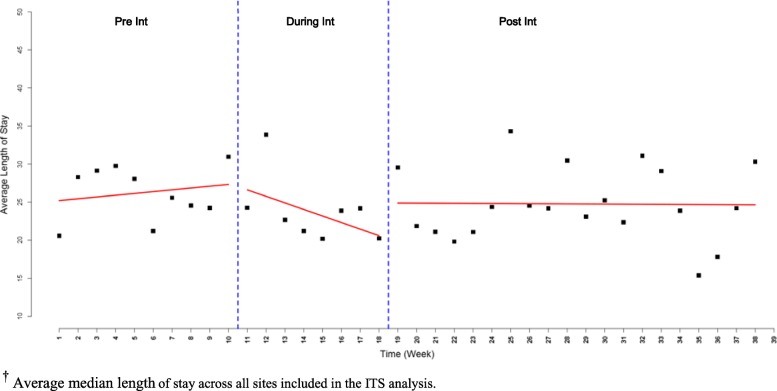
Change in length of stay

Data on LOS were not available for one of the six sites. Across the 5 remaining sites, the median LOS during the intervention period was four days shorter on average compared to the pre-intervention period, where a median LOS of 26.24 days (95% CI 23.67–28.80) was observed pre-intervention compared to 23.81 days (95% CI 20.13–27.49) during the intervention and 24.69 days (95% CI 22.43–26.95) post-intervention. Investigating the trend of median LOS over time using ITS analysis, there was an increasing trend in LOS in the pre-intervention period (Fig. 3); a decreasing trend in median LOS was observed during intervention implementation followed by a slight decreasing trend in LOS in the post-intervention phase. Patients were in hospital an estimated 4.35 fewer days (95% CI -18.08 – 9.39 *P-value* = 0.5351) in the post-intervention period compared to pre-intervention, however, the difference was not statistically significant. There was a decreasing trend in LOS both during and post-intervention. Upon visual inspection of the data, it appears that a decreasing trend in LOS corresponded with an increasing trend in mobilization. A decreasing trend (slope difference = − 0.65, 95% CI -2.19 – 0.88, *P-value* = 0.4051) of LOS was observed during the last weeks of the study compared to the first weeks of the post-intervention period.

#### Hospital-specific results

There was heterogeneity in the trend of median LOS over time across the five participating sites [see Additional file 6]. For most of the sites (80%; four of five sites) a decrease in LOS during the intervention and/or post-intervention periods was observed. The rate of decrease in median LOS was different across sites. No statistically significant differences were found in discharge destination [see Additional file 7].

## Discussion

In replicating the MOVE program in different hospital unit types, we found that the intervention had a positive effect on mobilization rates immediately following the eight week intervention. Three out of the seven hospitals demonstrated sustained mobilization rates following the post-intervention period. These three hospitals had strong senior leadership buy-in and staff engagement. One site was able to scale-up the MOVE initiative as a corporate priority while the other two sites were able to attain high staff involvement and central team support in adapting and implementing strategies. In contrast, four sites were unable to sustain mobilization efforts after the post-intervention phase. Of these other four sites, a number of factors (e.g. infectious outbreaks) impeded sustainability; two sites had a lack of senior leadership support and staff engagement from the beginning; and the last site included psychiatry/behavioural neurology units that provided long-term care. These latter units had high baseline mobility rates (74.5%), which left little room for improvement.

Ioannidis has written extensively on the need to replicate primary research [26–28] . This concept should also be applied to implementation studies whereby there is a paucity of literature on replication in different settings. Our study represented an unique attempt to replicate the implementation of an early mobilization strategy across different hospitals and inpatient units. The findings of this study are consistent with our previous evaluation in which there was a significant increase in median weekly observed mobilization [intercept difference = 10.56% (95% CI 4.94–16.18 *P* < 0.001)] and a decreased LOS (intercept difference = − 3.45 days, 95% CI: [− 6.67,− 0.23], *P* = 0.0356) over the study period compared to pre-intervention [25]. It is also consistent with mobilization studies conducted in other types of units and with specific patient populations, for example older patients with pneumonia [45], with acute illness [46], and in the ICU [47]. To further understand replicability of MOVE, other groups are independently implementing it in the US and Australia. Failure to replicate study findings by other teams in varying settings is a common shortcoming in many areas of research, including basic science and clinical investigation [27].

### Implications for tailoring, scaling up, and spread

In addition to being a successful replication study, the current project was an example of scaling up an intervention. Scale up is the “vertical diffusion or deliberate, systematic approaches to increasing the coverage, range, and sustainability of services” [48]. In this study, we deliberately focused on scaling up MOVE. MOVE was not reliant on expensive technology or large investment of resources and instead used available resources aligned with unit needs.

There is little research on scaling up or tailoring evidence-informed interventions. For example, in a systematic review strategies for scaling up implementation interventions in primary care, just 14 studies of the 272 studies met inclusion criteria and most were before-and-after design [30]. A unique MOVE feature is how it is designed to be tailored to local context. Based on focus groups with frontline clinicians, we created a list of common barriers and facilitators to mobilization. Using evidence on implementation strategies [49, 50] and behaviour change theory [51, 52], we identified implementation strategies for each barrier and facilitator [40]. Each site prioritized the most relevant barriers. We then provided sites with a list of the appropriate implementation strategies. Sites were then able to use implementation strategy resources, adapt them, or create new ones. The most common adaptation was to change formatting, colours, and add hospital logos to existing resources. This approach allows for local tailoring to promote sustainability, while selecting implementation strategies based on evidence and theory.

### Strengths and limitations

The current study had several strengths. First, the primary outcome, mobilization, is an objective outcome measure. Second, the study included a large sample. Third, the funding was only used for project evaluation. All hospital resources were provided in-kind, which facilitated sustainability and spread. Fourth, all MOVE resources are publicly available for use.

A few limitations should also be noted. First, when using an ITS design, it is possible that historical or contextual factors are responsible for the increase in mobilization and decreased LOS. However, we saw the same trend in mobilization across 11 hospitals in the original study [25] and seven hospitals in the replication study. The intervention starting point (i.e., the months in which the intervention was delivered) varied across hospitals. The consistency in findings, despite delivering the intervention in different months, provides more support that MOVE is responsible for the changes in mobilization. Second, this study involved new units in hospitals that previously implemented MOVE. Some hospitals may have already started spreading MOVE, so the pre-intervention data may not have represented baseline, thereby underestimating the impact of MOVE. Third, audits only provide a snapshot of mobilization. It is possible that implementation strategies produced large increases in mobilization not captured during audit times. We were not able to capture duration of mobility given budget constraints. Video cameras or personal exercise monitoring devices could be considered in future studies of mobility, if budget allows. To optimize sustainability of mobility monitoring, knowledge users suggested use of audit since this was done for auditing of hand hygiene purposes on the hospital units and could be used for both purposes. Moreover, we completed a test accuracy study prior to implementation showing good accuracy of this measure (likelihood ratio 12). Fourth, due to budget limitations of our study, we did not examine other clinically relevant outcomes measures such as muscle strength, delirium, decubitus ulcers, venous thrombosis, and pneumonia. Randomized trials have shown these outcomes to be impacted with improved mobility. [4–6, 10–13]. Fifth, we did not identify a significant decrease in LOS although this was seen in our previously completed, larger implementation study. This difference may be due to sample size of the current study or other confounding factors such as provincial initiatives that contributed to decreased length of stay during this implementation study.

## Conclusions

We successfully implemented a mobilization intervention in several inpatient units that was tailored to context. Mobilization increased during and after the intervention and declined during the sustainability phase in some sites. This study lends further support to the impact of MOVE at increasing patient mobilization across multiple types of hospital units.

## Additional files

Additional file 1: Template for Intervention Description and Replication (TIDieR) checklist. (DOCX 32 kb)

Additional file 2: Implementation Activties Delivered. (DOCX 23 kb)

Additional file 3: Participant Responses to Readiness Assessment Questions. (DOCX 19 kb)

Additional file 4: Difference in proportion of mobility between during and pre-intervention (left), and between post- and pre-intervention (right). (DOCX 29 kb)

Additional file 5: Weekly visual audit results for proportion of patients out of bed for site excluded in overall ITS analysis. (DOCX 135 kb)

Additional file 6: Difference in median LOS between during and pre-intervention (left), and between post- and pre-intervention (right). (DOCX 27 kb)

Additional file 7: Discharge destination. (DOCX 15 kb)

## Abbreviations

ITS: Interrupted time series
LOS: Length of stay (LOS)
MOVE ON: Mobilization of Vulnerable Elders (MOVE) in Ontario
