# Supplementary material for: Can the effects of the mobilization of vulnerable elders in Ontario (MOVE ON) implementation be replicated in new settings: an interrupted time series design
Source: BMC Geriatr. 2019 Apr 5;19:99. doi: 10.1186/s12877-019-1124-0 (PMC6451288; doi:10.1186/s12877-019-1124-0)
Supplement: Supplementary file 3 — Participant Responses to Readiness Assessment Questions. (DOCX 19 kb) [file 12877_2019_1124_MOESM3_ESM.docx]

**Additional file 3: Participant responses to readiness assessment questions.**

| **Questions** | **Site A Mean (SD) n = 15** | **Site B Mean (SD) n = 6** | **Site C Mean (SD) n = 6** | **Site D Mean (SD) n = 15** | **Site E Mean (SD) n = 15** | **Site F Mean (SD) n = 62** |
| --- | --- | --- | --- | --- | --- | --- |
| 1. There is a strong evidence basis for this statement: Early mobilization will prevent functional decline in older patients admitted to hospitals. | 4.80 (0.41) | 4.00 (1.55) | 4.67 (0.52) | 4.60 (1.06) | 4.93 (0.27) | 4.65 (0.91) |
| 1. Your institution has identified mobilization of elderly patients as a change priority. | 3.40 (1.88) | 3.67 (1.03) | 4.50 (0.55) | 2.90 (1.73) | 3.64 (1.60) | 3.63 (1.43) |
| 1. Implementing an education strategy is an appropriate and feasible strategy to address your institution’s change priority. | 4.07 (1.27) | 4.00 (0.89) | 4.33 (0.52) | 3.80 (1.26) | 4.00 (1.04) | 3.95 (1.25) |
| 1. Your institution accepts and rewards innovation in clinical practice. | 3.40 (1.12) | 4.33 (0.82) | 4.33 (0.82) | 3.40 (1.06) | 3.79 (1.42) | 3.48 (1.26) |
| 1. Now is the right time for implementing change (i.e., it will not compete with other major change priorities at your institution). | 3.90 (1.09) | 3.33 (1.75) | 4.00 (0) | 3.67 (1.23) | 3.71 (0.99) | 3.33 (1.75) |
| 1. Your institution’s senior leadership/clinical management support culture/practice change. | 4.00 (1.41) | 4.00 (0.52) | 3.50 (1.76) | 3.67 (1.23) | 4.00 (0.96) | 3.66 (1.34) |
| 1. Your institution promotes teamwork/team building to solve clinical care problems. | 3.80 (0.77) | 4.00 (0.89) | 4.67 (0.52) | 3.53 (1.30) | 4.21 (0.70) | 3.76  (1.08) |
| 1. There are adequate resources to support change (e.g., financial, human, and equipment). | 2.13 (1.06) | 3.33 (0.82) | 3.17 (0.98) | 3.00 (0.85) | 2.67 (1.30) | 3.84 (1.06) |
| 1. Senior leadership/clinical management will provide dedicated time for staff to prepare for and implement change. | 3.20 (1.32) | 3.33 (0.52) | 3.33 (0.52) | 3.00 (0.76) | 2.83 (1.40) | 2.37 (1.24) |
| 1. Your institution will allow time for staff to attend training and education on mobilization. | 3.47 (1.19) | 3.67 (0.82) | 4.17 (0.41) | 3.33 (1.22) | 4.00 (0.43) | 3.03 (1.46) |
| 1. Your institution is willing to measure and assess progress and continuously improve processes. | 3.53 (1.25) | 3.83 (0.75) | 4.17 (0.41) | 3.27 (1.03) | 3.85 (0.55) | 3.24 (1.43) |
| 1. Your institution will reinforce and reward positive teamwork behaviours and improvements in processes. | 3.40 (1.40) | 3.33 (0.52) | 4.00 (0.63) | 2.87 (0.99) | 3.38 (0.96) | 2.97 (1.49) |
| **Overall Summary Score** | **3.59 (1.34)** | **3.76 (0.97)** | **4.07 (0.87)** | **3.41**  **(1.23)** | **3.71 (1.26)** | **3.45 (1.40)** |

Response scale: 5 = strongly agree; 4 = agree; 3 = neither agree nor disagree; 2 = disagree; 1 = strongly disagree
