# Supplementary material for: Can the effects of the mobilization of vulnerable elders in Ontario (MOVE ON) implementation be replicated in new settings: an interrupted time series design
Source: BMC Geriatr. 2019 Apr 5;19:99. doi: 10.1186/s12877-019-1124-0 (PMC6451288; doi:10.1186/s12877-019-1124-0)
Supplement: Supplementary file 5 — Weekly visual audit results for proportion of patients out of bed for site excluded in overall ITS analysis. (DOCX 135 kb) [file 12877_2019_1124_MOESM5_ESM.docx]

**Additional file 5: Weekly visual audit results for proportion of patients out of bed for site excluded in overall ITS analysis.**


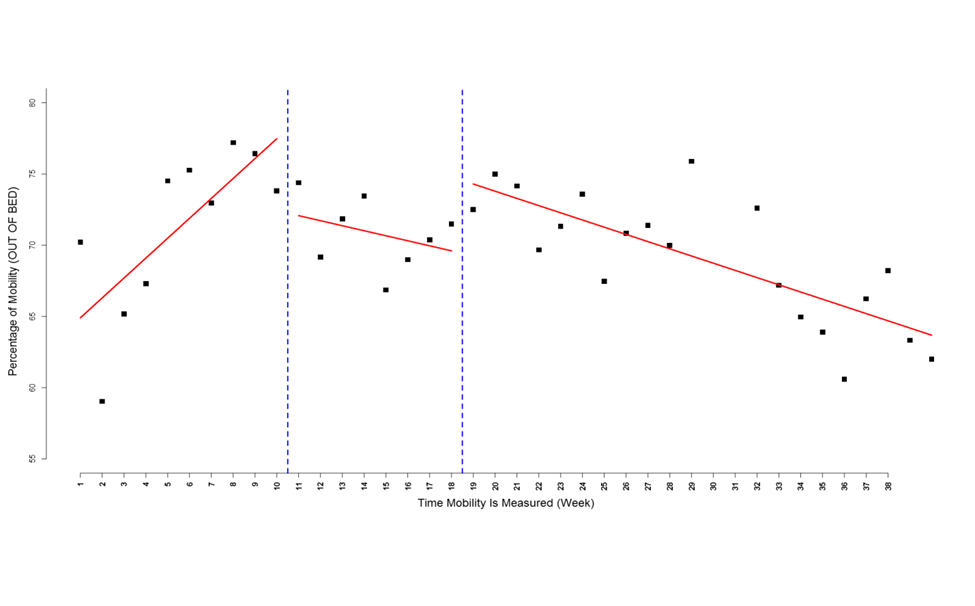


**Pre Int During Int Post Int**
