# Supplementary material for: Can the effects of the mobilization of vulnerable elders in Ontario (MOVE ON) implementation be replicated in new settings: an interrupted time series design
Source: BMC Geriatr. 2019 Apr 5;19:99. doi: 10.1186/s12877-019-1124-0 (PMC6451288; doi:10.1186/s12877-019-1124-0)

**Additional file 6: Difference in median LOS between during and pre-intervention (left), and between post- and pre-intervention (right).**


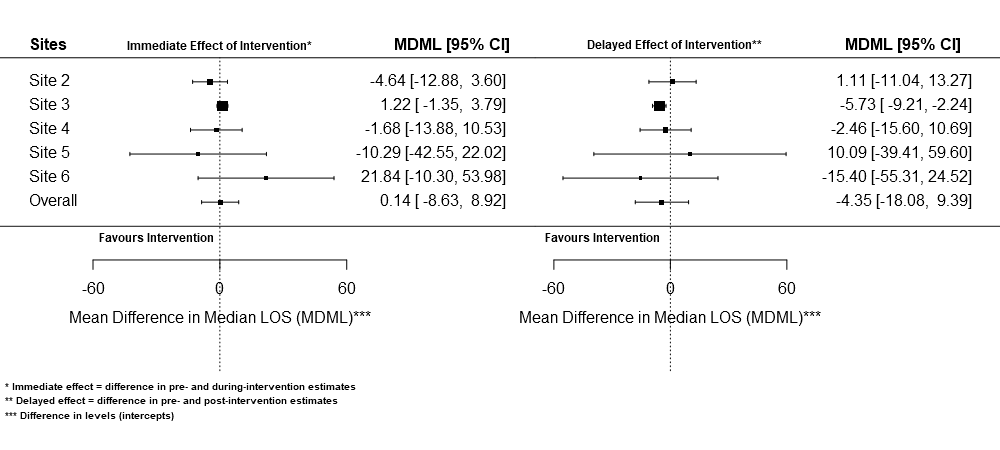

Supplement: Supplementary file 6 — Difference in median LOS between during and pre-intervention (left), and between post- and pre-intervention (right). (DOCX 27 kb) [file 12877_2019_1124_MOESM6_ESM.docx]
