# Supplementary material for: Can the effects of the mobilization of vulnerable elders in Ontario (MOVE ON) implementation be replicated in new settings: an interrupted time series design
Source: BMC Geriatr. 2019 Apr 5;19:99. doi: 10.1186/s12877-019-1124-0 (PMC6451288; doi:10.1186/s12877-019-1124-0)
Supplement: Supplementary file 7 — Discharge destination. (DOCX 15 kb) [file 12877_2019_1124_MOESM7_ESM.docx]

**Additional file 7: Discharge destination.**

| **Discharge Destination** | Percentage (95%CI) | | | |
| --- | --- | --- | --- | --- |
|  | Overall  n= (3098) | Pre-Intervention  n= (917) | During-Intervention  n= (535) | Post- Intervention  n= (1646) |
| Home | 55.94 (54.17, 57.70) | 55.83 (52.55, 59.07) | 58.13 (53.81, 62.33) | 55.28 (52.84, 57.70) |
| Rehab | 4.52 (3.84, 5.33) | 4.90 (3.64, 6.56) | 5.42 (3.72, 7.78) | 4.01 (3.14, 5.10) |
| Nursing Home | 18.11 (16.78, 19.52) | 18.43 (16.00, 21.13) | 15.70 (12.78, 19.13) | 18.71 (16.87, 20.70) |
| Acute Facility | 5.46 (4.69, 6.33) | 3.27 (2.26, 4.70) | 5.61 (3.88, 8.00) | 6.62 (5.49, 7.96) |
| Deceased | 2.71 (2.18, 3.36) | 3.60 (2.53, 5.07) | 3.74 (2.36, 5.81) | 1.88 (1.30, 2.70) |
| Other | 12.91 (11.76, 14.15) | 13.96 (11.81, 16.41) | 11.40 (8.90, 14.48) | 12.82 (11.26, 14.55) |
